# Un cas de volumineux Cystadénome Mucineux de l'ovaire au Centre Hospitalier Universitaire Régional De Ouahigouya (Burkina Faso)

**DOI:** 10.48327/mtsi.v2i2.2022.187

**Published:** 2022-06-20

**Authors:** Moussa SANOGO, Sansan Rodrigue SIB, OUÉDRAOGO, Issa OUÉDRAOGO, Korotimi SANOGO, Ibrahim SAWADOGO, Hyacinthe ZAMANÉ, Blandine BONANÉ

**Affiliations:** 1 Centre hospitalier universitaire régional (CHUR) de Ouahigouya, Burkina Faso; 2 Centre hospitalier universitaire Yalgado Ouédraogo, Ouagadougou, Burkina Faso

**Keywords:** Tumeur, Cystadénome mucineux, Ovaire, Laparotomie, Ouahigouya, Burkina Faso, Afrique subsaharienne, Tumor, Mucinous cystadenoma, Ovary, Laparotomy, Ouahigouya, Burkina Faso, Sub-Saharan Africa

## Abstract

**Introduction:**

Tumeur bénigne de l’âge moyen chez la femme, le cystadénome mucineux représente environ 20 % des tumeurs de l'ovaire. Il peut atteindre des tailles très importantes.

**Observation clinique:**

Nous rapportons le cas d'une patiente de 42 ans reçue pour une volumineuse masse abdomino-pelvienne montant jusqu'au niveau de l'appendice xiphoïde. L'imagerie a objectivé une formation liquidienne circonscrite occupant la cavité abdomino-pelvienne de 40,1 x 29 x 25,7 cm développée aux dépens de l'ovaire. Une laparotomie a objectivé un volumineux kyste de l'ovaire gauche. Une annexectomie gauche avec hémostase satisfaisante a été réalisée. La pièce opératoire pesait 13,5 kg. Les suites opératoires ont été simples. L'examen anatomo-pathologique a conclu à un cystadénome mucineux de l'ovaire.

**Conclusion:**

Le cystadénome mucineux de l'ovaire est une tumeur bénigne de l'ovaire pouvant atteindre des volumes très importants. Le traitement est chirurgical et les suites opératoires le plus souvent simples.

## Abstract (en)

**Introduction:**

A benign tumor of middle-aged women, mucinous cystadenoma accounts for about 20% of tumors of the ovary. It can reach very large sizes.

**Clinical observation:**

We report the case of a 42-year-old patient received for a voluminous abdomino-pelvic mass. The examination found a soft, rounded, fairly mobile abdomino-pelvic mass going up to the level of the xiphoid appendix with a light skin and collateral venous circulation. Imaging showed a circumscribed fluid formation occupying the abdomino-pelvic cavity of 40.1 x 29 x 25.7 cm developed at the expense of the ovary. A laparotomy brought to light a voluminous cyst at the expense of the left ovary with fluid content cowardly adhering to the abdominal wall and intimately to the left proboscis. The uterus and right adnexa were unremarkable. We performed a left adnexectomy with satisfactory hemostasis taking away the cyst. The adnexectomy piece weighed 13.5 kg. The surgical follow-ups were simple. Anatomo-pathological examination confirmed a mucinous cystadenoma of the ovary.

**Conclusion:**

Mucinous cystadenoma of the ovary is a benign tumor which can reach very large volumes. Its treatment is surgical and the follow-ups are usually simple

## Introduction

Le cystadénome mucineux ovarien est une tumeur bénigne qui se développe aux dépends de l’épithélium de surface de l'ovaire. Il s'agit d'une tumeur qui représente 15 à 20 % de toutes les tumeurs de l'ovaire [6]. Elle se rencontre généralement chez les femmes d’âge moyen, plus rarement dans les âges extrêmes de la vie [3]. Ces tumeurs sont très souvent unilatérales et peuvent atteindre des tailles très importantes. Nous rapportons un cas de cystadénome mucineux de 13,5 kg chez une patiente de 42 ans au CHUR de Ouahigouya.

## Observation Clinique

Patiente de 42 ans qui avait été reçue pour une volumineuse masse abdomino-pelvienne d’évolution progressive depuis un an dans un contexte d'aménorrhée. La patiente n'avait pas d'antécédent médical particulier. Elle aurait eu sa ménarche à 14 ans et le cycle menstruel était irrégulier. Elle n'avait aucune contraception. Sur le plan obstétrical, la patiente avait eu 8 grossesses, 8 accouchements, avec 6 enfants vivants et un antécédent de césarienne en 2003.

**Figure 1:**
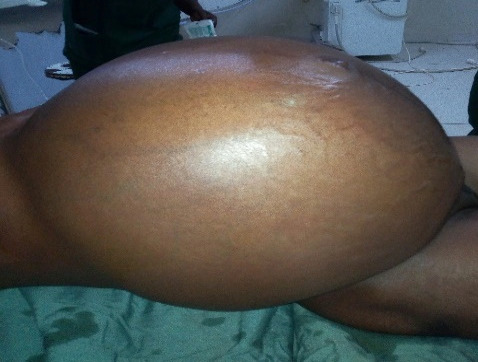
Aspect de I'abdomen avant la laparotomie Appearance of the abdomen before laparotomy

L'examen à l'entrée a trouvé une patiente amaigrie pesant 75 kg avec une perte de poids d'environ 10 kg. L’état général était assez bon et les conjonctives moyennement colorées. L'abdomen était volumineux, luisant avec une circulation veineuse collatérale et une cicatrice de césarienne. Le périmètre abdominal était de 120 cm (Fig. 1). La palpation abdominale montrait une masse abdomino-pelvienne molle, arrondie, assez mobile remontant jusqu'au niveau de l'appendice xiphoïde. Au spéculum, le col et les parois vaginales étaient d'allure saine. Les culs-de-sac étaient comblés et non douloureux.

L’échographie a objectivé une volumineuse masse abdomino-pelvienne à contenu liquidien sans pouvoir spécifier avec certitude son origine. La tomodensitométrie abdomino-pelvienne a objectivé une formation liquidienne circonscrite d'origine ovarienne occupant la cavité abdomino-pelvienne de 40,1 x 29 x 25,7 cm avec un effet de masse sur les voies excrétrices urinaires et une urétéro-hydronéphrose bilatérale d'amont prédominant à droite, sans signe d'agressivité régionale ou à distance.

**Figure 2:**
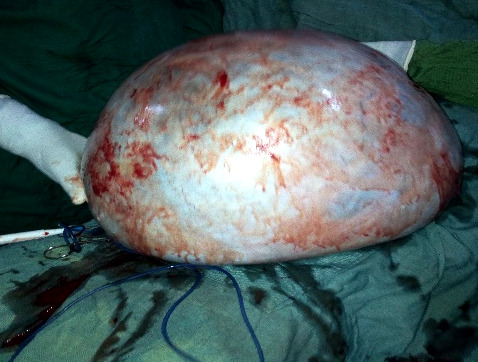
Aspect du kyste avant l'annexectomie Appearance of the cyst before adnexectomy

Au vu des résultats, nous avons proposé une laparotomie. Sous anesthésie générale, nous avons fait une incision médiane xypho-pubienne. Après la cœliotomie, nous avons aspiré une ascite d'environ 150 ml. L'exploration a mis en évidence un volumineux kyste de l'ovaire gauche à contenu liquidien (Fig. 2). La paroi du kyste adhérait faiblement à la paroi abdominale et à la trompe gauche de façon plus importante. L'utérus et l'annexe droite étaient sans particularité. Nous avons réalisé une annexectomie gauche emportant le kyste avec une hémostase satisfaisante. La pièce d'annexectomie pesait 13,5 kg. Nous avons mis en place un drain dans le Douglas.

En peropératoire la patiente a bénéficié de la transfusion d'une poche de culot globulaire rouge iso groupe iso rhésus. Le taux d'hémoglobine initiale était de 8 g/dl avec une spoliation sanguine de 300 ml. Les suites opératoires ont été simples. La patiente a été libérée à J5 avec une bonne cicatrisation observée à la surveillance du 45^e^ jour.

**Figure 3:**
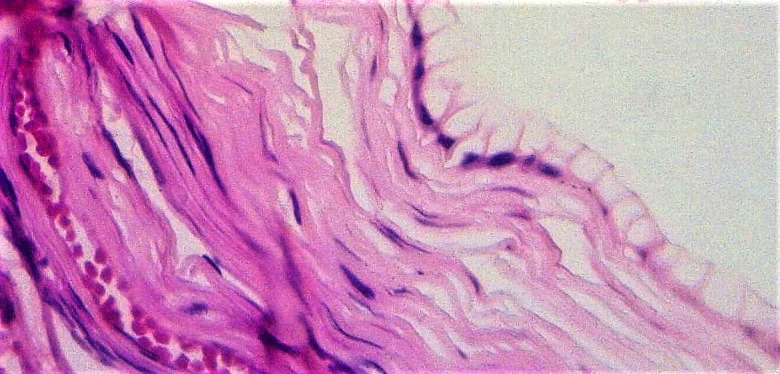
Examen anatomopathologique de la pièce opératoire. G400 coloration hématéine-éosine. Paroi kystique avec un épithélium cylindrique muco-sécrétant reposant sur un chorion fibreux, congestif Anatomo pathological examination of the operating piece. G400 hematein-eosin staining. Cystic wall with a muco-secreting cylindrical epithelium resting on a fibrous, congestive chorion

L'examen anatomo-pathologique a décrit une pièce de kystectomie pesant 13,5 kg et mesurant 40 × 28 × 28 cm avec une portion de trompe accolée qui mesurait 3 × 1 cm. La surface externe était lisse, blanc-grisâtre. À la coupe, la pièce était kystique, multiloculaire à contenu gélatineux, liquide, filant. On notait une absence de végétation et de zone de renflement intra-kystiques. Il n’était pas retrouvé de parenchyme ovarien résiduel. À la coupe de la trompe, la lumière était libre. Les coupes montraient des parois kystiques revêtues par un épithélium cylindrique simple muco-sécrétant dont les cellules possédaient un noyau refoulé au pôle basal et un cytoplasme clair, chargé de mucus. Le chorion était conjonctif, dense, fait de fibroblastes et de fibres collagènes (Fig. 3).

Une surveillance clinique postopératoire a été instituée tous les trois mois jusqu’à 1 an sans qu'aucune complication ne soit observée.

## Commentaires

Le cystadénome mucineux de l'ovaire est fréquent (15 à 20 % des tumeurs ovariennes). Dans la littérature, il ressort que les volumineux cystadénomes mucineux de l'ovaire sont plus fréquents entre 30 et 50 ans [3]. Des auteurs ont décrit des cas chez des patientes plus jeunes (13-29 ans) [1, 4, 7]. L'apparition de cette tumeur au-delà de 50 ans pourrait être un facteur péjoratif faisant craindre la malignité.

Ces tumeurs peuvent atteindre des tailles très importantes avec un poids allant de 7,2 à 9,2 kg [4]. Dans le cas que nous présentons, la taille était de 40 x 28 x 28 cm et le poids de 13,5 kg.

Le diagnostic peut être évoqué par l’échographie et la tomodensitométrie abdomino-pelvienne. Ce diagnostic dans notre contexte est souvent difficile du fait de l'inaccessibilité géographique et financière des examens d'imagerie médicale. Les patientes sont souvent obligées de faire de longues distances pour avoir accès à des examens d’échographie et surtout de tomodensitométrie. Lorsque ces examens sont disponibles, le coût n'est pas accessible à toutes.

Certains marqueurs tumoraux (surtout pour une surveillance postopératoire) peuvent être demandés pour se rassurer de la nature bénigne de la tumeur [2]. Dans notre cas, les arguments cliniques et paracliniques étaient en faveur d'une tumeur bénigne. Nous n'avons pas dosé les marqueurs tumoraux parce que cet examen biologique n’était pas disponible. L’échographie n’était pas très concluante comme rapporté par certains auteurs [2]. La tomodensitométrie a permis d'orienter le point de départ de la tumeur et de rassurer sur l'absence de signes péjoratifs en rapport avec la malignité.

À un stade de début, avant de devenir très volumineuse avec des complications, la tumeur peut simuler une grossesse chez certaines patientes [2], surtout si un désir de grossesse existe. Notre patiente a pensé dans un premier temps être enceinte. Elle aurait consulté des accoucheuses villageoises puis même entrepris une consultation prénatale dans une structure périphérique où aucun examen complémentaire n’était réalisable. Vu l'augmentation importante du volume de l'abdomen, la patiente a été orientée vers le CHUR situé à une centaine de kilomètres de son lieu de résidence.

En dehors de la grossesse, lorsque l'abdomen est volumineux, le diagnostic de fibrome utérin ou d'ascite est souvent évoqué dans notre contexte. Chez certaines patientes, une ponction du kyste est même effectuée en tant qu'ascite et adressée au service de gastro-entérologie [2].

L'examen gynécologique montre le caractère pelvien de la tumeur surtout lorsque l'augmentation du volume de l'abdomen est associée à des troubles du cycle et à des douleurs pelviennes.

La fréquence élevée des myomes et des pathologies gastriques avec ascite tend à orienter vers ces diagnostics. Il se pose alors le problème de la disponibilité d'agents de santé qualifiés.

La prise en charge est chirurgicale. Il s'agit d'une kystectomie ou souvent d'une annexectomie lorsque toute l'annexe est confondue au kyste à cause des adhérences comme c’était le cas chez notre patiente ou lorsqu'il y a une torsion d'annexe. La voie cœlioscopique n'est pas indiquée du fait de la taille des tumeurs [7].

Sur le plan histopathologique, selon la classification de l'OMS, il s'agit d'une tumeur du revêtement épithélial de l'ovaire, caractérisée par une prolifération des cellules muco-sécrétantes. Elles peuvent être bénignes (80%), malignes (10%) ou frontières (10%).

## Conclusion

Les tumeurs ovariennes peuvent atteindre des tailles considérables. C'est le cas du cystadénome mucineux de l'ovaire. Les examens radiologiques permettent d'orienter vers le diagnostic qui est confirmé par l'examen anatomo-pathologique.

La prise en charge est chirurgicale et dépend de l’âge de la patiente et de la taille de la tumeur. Une surveillance postopératoire s'impose même en dehors de facteur péjoratif.

## Liens D'intérêts

Les auteurs ne déclarent aucun lien d'intérêt.

## Contribution Des Auteurs

Moussa SANOGO, Sansan Rodrigue SIB et Salam OUÉDRAOGO ont participé à la prise charge de la patiente. Ils ont rédigé la première version du manuscrit. Korotimi SANOGO, Ibrahim SAWADOGO, Issa OUÉDRAOGO, Yacinthe ZAMANÉ, Blandine BONANÉ ont apporté des contributions essentielles au document. Moussa SANOGO et Sansan Rodrigue SIB ont coordonné la rédaction du manuscrit.
